# Transcriptome Variability in Keratocystic Odontogenic Tumor Suggests Distinct Molecular Subtypes

**DOI:** 10.1038/srep24236

**Published:** 2016-04-12

**Authors:** Shijia Hu, Kimon Divaris, Joel Parker, Ricardo Padilla, Valerie Murrah, John Timothy Wright

**Affiliations:** 1 Pediatric Dentistry, University of North Carolina, Chapel Hill, NC, USA; 2 Faculty of Dentistry, National University of Singapore, Singapore; 3 Epidemiology, Gillings School of Global Public Health, University of North Carolina-Chapel Hill, Chapel Hill, NC, USA; 4 Cancer Genetics, University of North Carolina-Chapel Hill, Chapel Hill, NC, USA; 5 Diagnostic Sciences, School of Dentistry, University of North Carolina-Chapel Hill, Chapel Hill, NC, USA

## Abstract

Keratocystic Odontogenic Tumor (KCOT) is a locally aggressive developmental cystic neoplasm thought to arise from the odontogenic epithelium. A high recurrence rate of up to 30% has been found following conservative treatment. Aggressive tumor resection can lead to the need for extensive reconstructive surgery, resulting in significant morbidity and impacting quality of life. Most research has focused on candidate-genes with a handful of studies employing whole transcriptome approaches. There is also the question of which reference tissue is most biologically-relevant. This study characterizes the transcriptome of KCOT using whole genome microarray and compare it with gene expression of different odontogenic tissues (“dentome”). Laser capture microdissection was used to isolate the neoplastic epithelial tissue in 20 cases. KCOT gene expression was compared with the “dentome” and relevant pathways were examined. Cluster analysis revealed 2 distinct molecular subtypes of KCOT. Several inflammatory pathways were activated in both subtypes. The AKT pathway was activated in one subtype while MAP kinase pathway was activated in the other. Additionally, *PTCH1* expression was downregulated in both clusters suggesting involvement in KCOT tumorigenesis. In conclusion, this study provides new insights into the transcriptome of KCOT and highlights pathways that could be of diagnostic and prognostic value.

In 2005, the World Health Organization reclassified the Keratocystic Odontogenic Tumor (KCOT) from a cyst to a tumor to better reflect its neoplastic nature^1^. KCOT is a benign but locally aggressive developmental cystic neoplasm historically thought to arise from the odontogenic epithelium^2^ and frequently is associated with the follicle of unerupted teeth. During the last decade there has been a resurgence of interest in and efforts to understand tumorigenesis of KCOTs with the ultimate goal of developing better diagnostic and treatment approaches.

Despite advancements in antineoplastic therapies, surgical intervention remains the treatment of choice for KCOTs. A high recurrence rate of up to 30% has been found following conservative treatment such as enucleation and curettage, creating a challenge in determining the optimal extent of surgical resection. Nevoid basal cell carcinoma syndrome (NBCCS) is a disorder that presents with multifocal KCOT; these cases have even higher recurrence rates when compared to isolated unifocal cases not associated with NBCCS^3^. Aggressive tumor resection can lead to the need for extensive reconstructive surgery and rehabilitation for patients with KCOTs, causing significant morbidity and negatively impact their quality of life.

Little is known about the molecular profile of KCOTs; however, recent reports have shed some light on molecular pathways driving the tumorigenesis of KCOT. For example, the PTCH1 pathway has been the primary focus of candidate-gene studies due to its association with the NBCCS^4^,^5^,^6^. Other potential aberrant pathways including SHH^7^, WNT signaling^8^, p53^9^ and matrix metalloproteinases^10^ are of interest. These candidate-gene studies aid in the advancement of precision medicine^11^ for improved diagnostics for predicting prognosis and managing KCOT. However, only 2 published studies to date have used next-generation approaches including genomics and transcriptomics methods to characterize the tumorigenesis of KCOT. In a 2007 report, Heikinheimo *et al.* used a 588 cancer-related human cDNA array^12^ for the study of KCOT and more recently they employed a more extensive whole genome array^13^. These studies provided valuable initial insights into the molecular basis of KCOT development.

Unraveling the molecular basis of KCOT can help identify altered pathways involved in its tumorigenesis, as well as discover potentially informative cell markers for its diagnosis and treatment. Thus, the objective of this study was to characterize the transcriptome of KCOTs using a whole genome microarray and compare it with the expression profile of a biologically-relevant odontogenic tissue (referred to as “dentome”)^14^.

## Methods

### Patient recruitment and sample collection

The study was conducted in accordance with approved human subject research guidelines and was approved by the local institutional review board and the ethics committee of the University of North Carolina-Chapel Hill. Patients diagnosed with isolated KCOT and scheduled for tumor removal at the Department of Oral and Maxillofacial Surgery at the University of North Carolina-Chapel Hill between 2005 and 2008 formed the study sample. All participants (N = 20) provided written informed consent. Diagnosis of KCOT was confirmed by a board-certified oral pathologist and another investigator using the 2005 WHO Histologic Classification of Odontogenic Tumors. Patients presenting with NBCCS were excluded from this study. Six fresh frozen samples were obtained during the surgical procedures and 14 formalin-fixed paraffin-embedded (FFPE) samples were obtained from the Department of Oral and Maxillofacial Pathology at UNC-Chapel Hill after their histological diagnosis was confirmed. Potential associations of gene expression with participants’ demographic characteristics including gender, age, race, and tumor recurrence were examined using bivariate methods (Fisher’s exact test) and a conventional p < 0.05 statistical significance criterion.

### Sample preparation

All 20 samples were decalcified for 1–4 weeks using a solution containing water, hydrochloric acid, EDTA, tetrasodium tartrate and potassium sodium tartrate (Richard Allan Scientifics). The samples were then sectioned at −35 °C at a thickness of 7 microns and lightly stained with hematoxylin and eosin. These slides were then used for the microdissection of target tumor epithelial cells.

**Figure 1:**
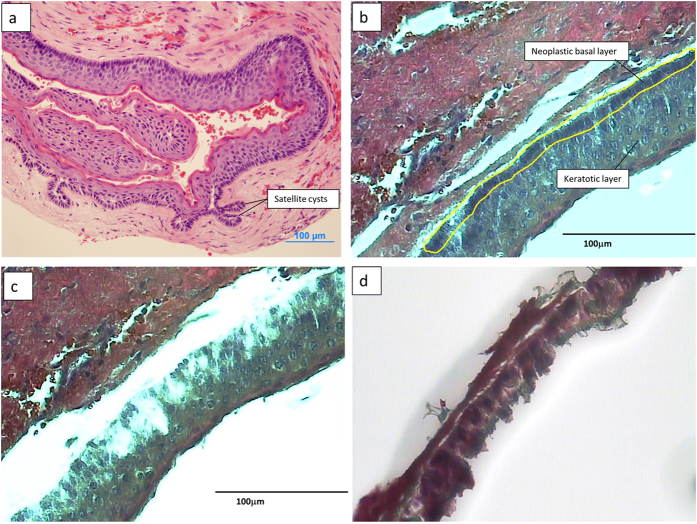
The micrographs show the laser capture of the epithelial portion of a KCOT sample. (**a**) light micrograph of KCOT at 4X showing formation of satellite cysts, (**b**) laser capture outline of epithelial cells showing target basal epithelial cells, (**c**) remnants of the stroma tissue after LCM and (**d**) captured cells on Capsure cap. Scale bar: 100 μm.

Laser capture microdissection (LCM)^15^ is a technique that allows the isolation of tissue that is 1 cell thick. KCOT is a cystic tumor containing loose surrounding stroma and a keratotic layer that arises from a neoplastic basal layer with numerous satellite cysts (Fig. 1a,b). LCM allowed isolation of discrete areas of the basal neoplastic layer (Fig. 1c,d) for the examination of a purer cell population. The technique has been successfully used in microarray studies for both fresh^16^ and formalin fixed samples^17^. The AutoPix^TM^ automated system (Arcturus Engineering, Santa Clara, CA, USA) was used for LCM. The captured cells were pooled for each sample and placed in RNA extraction buffer. RNA was isolated from the tumor cells using the PicoPure RNA Isolation kit (Arcturus Bioscience, Santa Clara, CA, USA). The quality and yield of total RNA were assessed using the Agilent Bioanalyzer 2100 (Agilent Technologies, Palo Alto, CA, USA).

### Microarray analysis

Agilent whole genome human oligonucleotide microarrays which contain 44 thousand 60-mer oligonucleotides representing over 41 thousand probes and transcripts was used for whole transcriptome analysis. The Human Universal Reference RNA from Stratagene (Santa Clara, CA, USA) served as a control to standardize hybridization levels between microarrays and was coupled to Cy5. Two hundred nanograms of total RNA from the KCOT samples were converted into labeled cRNA with nucleotides coupled to fluorescent dye Cy3 using the Low RNA Input Linear Amplification Kit (Agilent Technologies, Palo Alto, CA). The Cy3-labeled cRNA (1.65 ng) from each sample and Cy5 coupled universal reference RNA was hybridized to whole genome array formatted chips. The hybridized array was washed, scanned and data extracted from the scanned image using Feature Extraction version 9.5 (Agilent Technologies, Palo Alto, CA).

The microarray data were submitted to the Gene Expression Omnibus (GEO) microarray database (accession number GSE68532).

### Determination of reference tissue and bioinformatics analysis

Human odontogenic tissue whole transcriptome data, also known as the “dentome” was used to help identify the most appropriate (based on being the most similar) odontogenic reference tissue to be used for comparison with the tumor samples. As part of earlier work conducted by our group, multiple embryonic teeth were used to obtain 4 samples each of human odontoblasts, pre-secretory ameloblasts and secretory ameloblasts using microdissection to isolate discrete samples of the different cell types and development stages from which gene expression data were obtained. (Gene Expression Omnibus microarray database accession number GSE63289)^14^. Multiclass significance analysis of microarrays 4.0 (SAM) was conducted between the 3 types of normal odontogenic tissue and 60 genes were found to be expressed differentially at a FDR of <20%. A cluster analysis, using these 60 genes that differentiated the normal odontogenic tissue, was conducted using Cluster 3.0 between the KCOT samples and the normal odontogenic tissues. Results of this analysis between normal odontogenic tissues and KCOT were then visualized using Java TreeView-1.1.6r2 to establish the normal tissue with a gene expression profile most similar to KCOT.

Gene set enrichment analysis (GSEA)^18^ was conducted with GSEA v2.1.0 from the Broad institute (Cambridge, MA, USA) using the Molecular Signatures Database (MSigDB) curated gene set^19^ “all curated gene sets v4.0” and 1000 permutations. Differential expression between the tumor and comparison normal tissue was calculated with SAM. Genes of interest were carried forward and interrogated using the Ingenuity pathway analysis (IPA), including canonical pathway and upstream analyses. Upstream analysis is a model that predicts activation or inhibition of upstream regulators based on the expression levels of downstream molecules. The model has the advantage of detecting genes with possible gain-of-function mutation that did not show an increase level of expression.

### Microarray data validation via NanoString analysis

The NanoString nCounter system (Seattle, WA, USA) was used to validate the microarray gene expression data. The nCounter system is based on a direct multiplexed measurement of gene expression and offers high levels of precision and sensitivity^20^. The nCounter Human Cancer Reference codeset (http://www.nanostring.com/products/gene_expression_panels) which profiles 230 cancer-related human genes and 6 internal reference genes was used. In this analysis, the microarray data of a subset of 3 KCOT and 2 normal secretory ameloblast samples were examined. Hybridization reactions were performed using 50 ng of total RNA with reporter and capture probes, in accordance to the manufacturer’s instructions. The NanoString nCounter digital count readings were extracted, normalized and analyzed using nSolver v2.5 to obtain fold-change values between KCOT and normal tissue. These data were then compared to the corresponding KCOT and normal tissue microarray fold change values. A scatterplot was constructed using the 20 most upregulated and downregulated genes in the nanoString analysis between KCOT and secretory ameloblast with differential expression from the microarray data (Supplementary Table 1). The 2 sets of data showed good correlation (r-0.65) between the microarray and nanoString data for genes showing the highest fold change differences (Supplementary Fig. 1).

## Results

RNA extracted from the LCM samples had a 260/280 ratio of between 2.17 and 2.20 and a yield of between 914 ng to 1225 ng per sample, demonstrating reasonable quality and yield.

### Reference tissue and multiclass analysis

**Figure 2:**
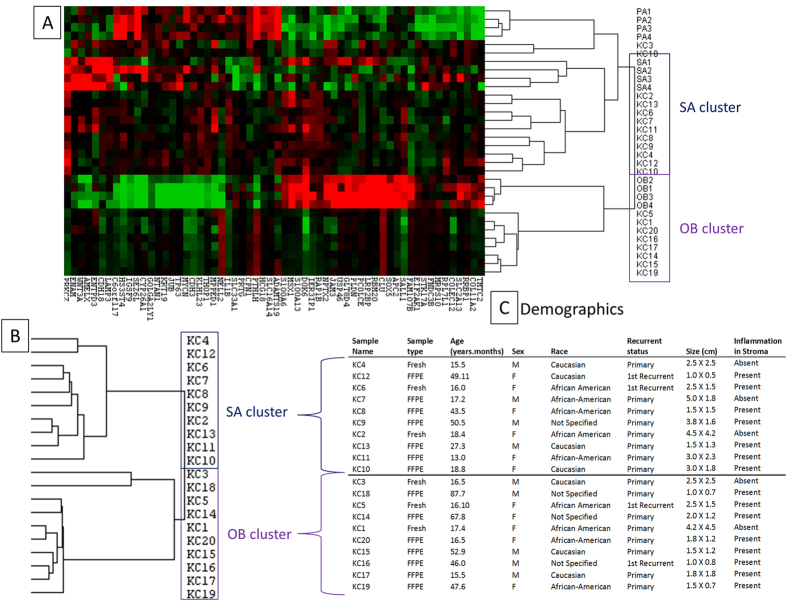
Cluster analysis to determine reference tissue. (**A**) Heat map of the 3 different odontogenic tissues (OB – Odontoblast, PA – Pre-secretory ameloblast, SA – Secretory ameloblast) and KCOT (KC) clustered using the 60 odontogenic tissue defining genes. (**B**) Array tree showing 2 clusters of the 2 KCOT molecular subtypes (odontoblast and secretory ameloblast clusters). (**C**) Demographics and tumor recurrence.

When cluster analysis was conducted with the 60 odontogenic tissue defining genes, the KCOT samples appeared to cluster into 2 distinct molecular subtypes (Fig. 2A). One of the subtypes clustered with secretory ameloblast (SA) while the other clustered with odontoblast (OB), as such the clusters were designated as secretory ameloblast-like KCOT (sKC) and odontoblast-like KCOT (oKC) respectively. In order to confirm the finding of 2 distinct subtypes, an unsupervised cluster analysis of all the genes were performed with the 20 KCOT samples. The tumor samples separated into 2 distinct subtypes as shown in the cluster tree (Fig. 2B). The Fisher exact test did not show significant association of the clusters (Fig. 2C) with race, recurrence, gender, tumor type, age and size of tumor. The level of inflammation in the stroma was graded as being present or absent (Supplementary Fig. 2). There was no significant association of the clusters with the presence or absence of inflammation in the stroma.

**Figure 3:**
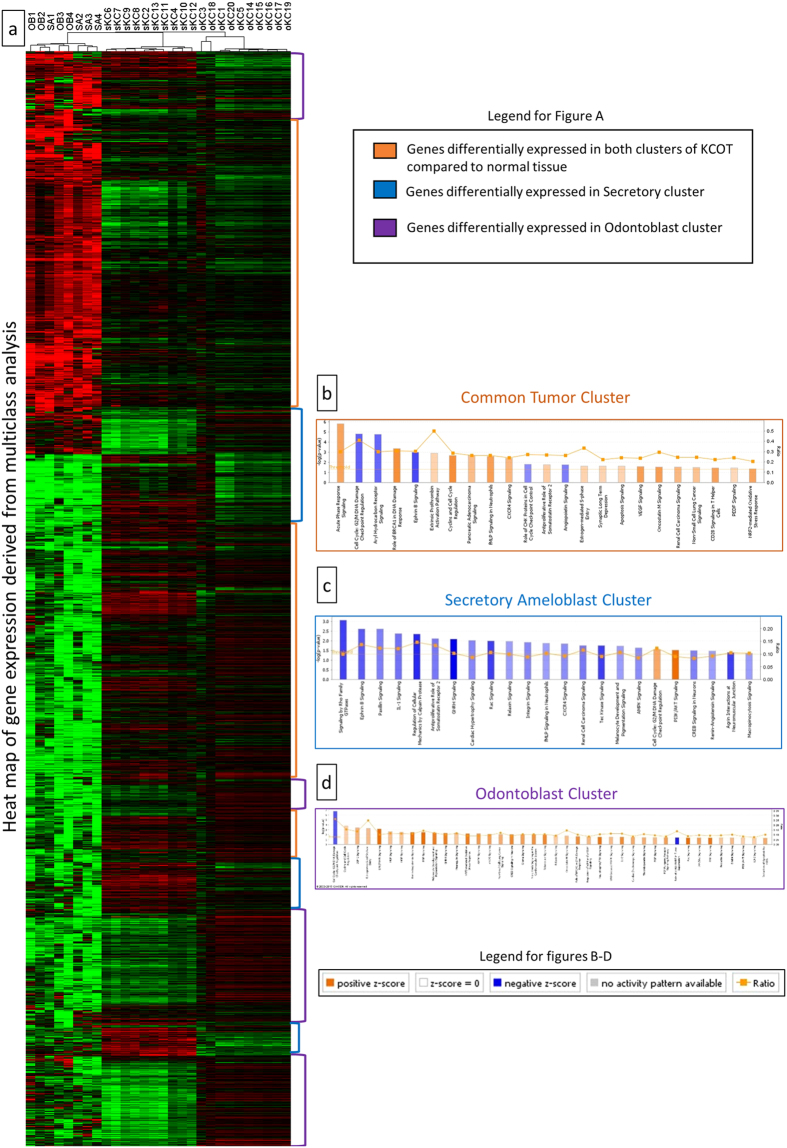
Multiclass analysis and pathway analysis of the different tumor clusters. (**a**) Heat map of the genes with a FDR <1% that are differentially expressed in the 4 clusters (OB – odontoblast, SA – Secretory Ameloblast, oKC – odontoblast-like KCOT, sKC – secretory ameloblast-like KCOT) from a SAM multiclass analysis. The color bars on the right of the heat map shows the groups of genes used to define each cluster’s differential gene expression. (**b**) Canonical pathways that are differentially expressed for the common tumor cluster in IPA. (**c**) Canonical pathways that are differentially expressed for the secretory ameloblast cluster in IPA. (**d**) Canonical pathways that are differentially expressed for the odontoblast cluster in IPA.

The discovery of 2 distinct molecular clusters of KCOT did not allow the designation of a single normal odontogenic tissue for gene expression comparison, as such, a multiclass analysis approach was employed. SAM multiclass analysis of genes differentially expressed at a FDR <1% was conducted between the 2 KCOT clusters (sKC, oKC) and 2 associated normal tissues (SA, OB) (Fig. 3a). The gene expression data were analyzed in 3 clusters. The “common tumor cluster” consists of 3166 genes which were differentially expressed in the 2 tumor clusters (sKC, oKC) compared to the 2 normal tissue clusters (OB, SA). The “secretory ameloblast cluster” consists of the 985 differentially expressed genes in the sKC cluster compared to the other 3 groups. The “odontoblast cluster” consists of 902 differentially expressed genes in the oKC cluster compared to the other 3 groups (Supplementary Table 2). The fold changes were then used for pathway and GSEA analyses.

### Canonical pathways and gene set enrichment analysis

Ingenuity pathway analysis was used to examine the activated and inhibited canonical pathways for each tumor cluster. Canonical pathways with an absolute z-score >1 and *p*-value < 0.05 are presented in Table 1 showing the pathways exhibiting the greatest activation/inhibition that were significantly different.

**Figure 4:**
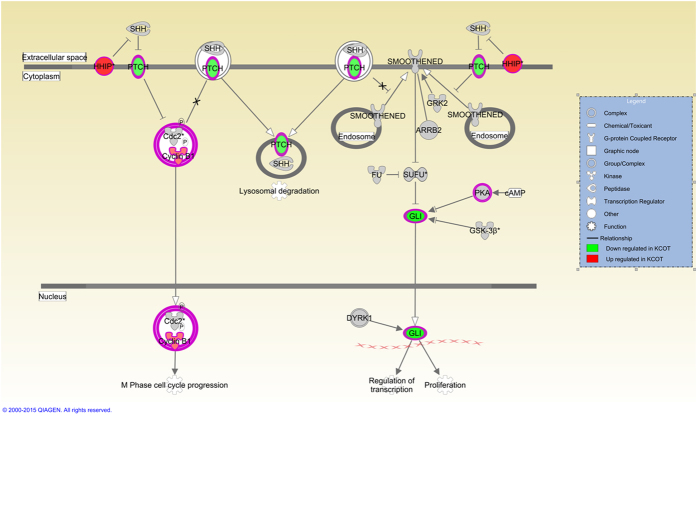
Sonic hedgehog signaling pathway. The upregulated and downregulated molecules of the pathway is shown according to the legend.

The common tumor cluster had 18 activated and 5 inhibited pathways (Fig. 3b). There were 7 pathways involved in cell cycle regulation. Notably, 6 Inflammatory (Immune) response/cytokine signaling pathways were found to be differentially expressed in addition to 5 Cellular growth, proliferation and apoptosis pathways and 3 Cancer pathways. GSEA conducted between the common tumor cluster and normal tissues showed that 14 gene sets were significantly enriched (nominal p-value < 0.05) and 1 gene set was inhibited (nominal p-value < 0.05) (Table 2). Specifically, the Sonic hedgehog signaling (SHH/PTCH1) pathway was not found to be significantly differentially expressed between normal and tumor tissue. Closer examination of the SHH/PTCH1 pathway showed downregulation of *PTCH1* and its downstream target *GLI* without changes to *SHH* (Fig. 4).

The sKC cluster had 2 activated and 20 inhibited pathways (Fig. 3c). All Inflammatory (Immune) response/cytokine signaling pathways and most of the Cellular growth, proliferation and apoptosis pathways were found to be inhibited except for the PI3K/AKT Signaling pathway.

The oKC cluster had 38 activated and 2 inhibited pathways (Fig. 3d). Most of these pathways involved Cellular growth, proliferation and apoptosis. Activation of MAP kinase-related pathways were also noted.

### Upstream analysis

Table 3 presents predicted differentially activated genes with *p*-values < 0.05 along with the corresponding z-scores. In the common tumor cluster, 21 molecules were predicted to be inhibited and 57 to be activated when compared to normal tissue. These upstream molecules were mainly transcription regulators (21 molecules), cytokines (10 molecules) and kinases (8 molecules). Two molecules were predicted to be activated in the secretory ameloblast tumor sKC cluster, namely the complex *Cg* and cytokine *CSF2*. In addition, 3 transcription regulators and 2 kinases were predicted to have differential activity in the odontoblast cluster.

## Discussion

A major finding of this study was the discovery of 2 distinct clusters of KCOT that exhibit similar phenotype despite differences in molecular pathway activity. In addition, *PTCH1* and *GLI* expression were found to be downregulated in both clusters suggesting involvement in the tumorigenesis of non-NBCCS associated KCOT. Moreover, the study provides a comprehensive characterization of KCOT transcriptome that can serve as a hypothesis-generating resource for the advancement of precision medicine for the diagnosis and treatment of KCOT.

The finding of 2 distinct molecular clusters was in contrast to the findings of Heikinheimo *et al.* who described a more homogeneous profile of KCOT^13^. However, comparing the findings of the 2 studies is difficult due to the use of gingiva tissue as the comparison tissue in their study. In the present study we interrogated the human “Dentome” to determine what tissue was most similar in gene expression profile to KCOT. It remains unclear what would be the optimal comparative oral tissue to use to help delineate differential gene expression in KCOT or other odontogenic tumors. Our use of a universal RNA allows for the normalization between arrays within the study and also can be used across studies which employ the universal RNA as an internal standardization that allows better comparison from one array to another. Fisher-exact tests did not find any association between the molecular clusters and the recurrence status and size of the tumor. Unfortunately, in this cross-sectional study, the recurrence status may not be accurate due to a lack of long term follow up and the aggressiveness of the tumor annot be assessed by size alone as time of diagnosis plays a role in tumor size in addition to aggressiveness. Inherent to the study of tumors in humans is the inability to access the growth of tumors over time as they are removed as soon as they are discovered.

The present study did find that most KCOT showed gene expression profiles that more closely resemble secretory ameloblast which is a differentiated cell type of the dental lamina from which KCOT is thought to arise. However, it is surprising that a smaller subset of tumors showed a gene expression profile more closely associated with odontoblast that is derived from a mesenchymal cell lineage. Differences in the 2 molecular subtypes includes activation of PI3K/AKT Signaling in sKC and activation of the MAP kinase pathway in oKC. Earlier studies have found activation of the AKT pathway in KCOT^21^ which have been extensively targeted in cancers for therapy^22^ and may provide a novel treatment modality for this particular subtype of KCOT. Conversely, the oKC subtype had activation of 3 MAP kinase associated pathways. The MAP kinase pathway has been implicated in KCOT development^23^. In addition, the oKC subtype showed activation of numerous Cellular growth, proliferation and apoptosis pathways that were not seen in the sKC subtype including the JAK/STAT pathway. Recently, a trial targeted this pathway in solid tumors with good results^24^ which could be explored as an adjunctive therapy before surgery to reduce the size of the tumors and thus extent of resection and reconstruction needed to reduce the high recurrence rate of KCOT.

The canonical pathway that showed the biggest difference in the “common tumor cluster” was the activation of the Acute Phase Response Signaling pathway. Additionally, 5 other inflammatory pathways were activated in the common tumor cluster compared to the normal tissue. Furthermore, upstream analysis predicted the activation of several pro-inflammatory markers. GSEA also showed several inflammatory gene sets that were enriched in KCOT. Other investigators^4^,^25^ also described the presence of inflammation around KCOT. As such, inflammatory pathways should be further investigated in the context of KCOT. Interestingly, both molecular clusters were found similar in the presence or absence of inflammation in the stroma determined histologically, suggesting that the activated inflammatory pathways may be independent of the inflammatory status of the tumor.

In addition, several cell cycle mechanism pathways and GSEA gene sets were found to be disturbed, corresponding to the uncontrolled proliferation found in the neoplasm. Upstream analysis also predicted inhibition of *CDKN1A* which has been implicated heavily in DNA damage response^26^ and control of cell cycles to prevent proliferation of neoplastic cells. Other notable upstream molecules predicted to be upregulated include members of the MAP kinase pathways such as *JUN*, *MAP3K14*, *ERK* and *JNK*, all of which have been heavily implicated with the development of cancers^27^.

The SHH/PTCH1 pathway which is known to cause NBCCS (OMIM # 10940), was specifically interrogated. Individuals with NBCCS have *PTCH1* mutations and presents clinically with multiple and recurrent KCOT as well as other neoplasms (e.g. basal cell carcinoma). Historically, mutations in the *PTCH1* gene were found in more than 85% of syndromic KCOT but less than 30% of sporadic cases. More recent studies suggest that the proportion of sporadic KCOT with *PTCH1* mutations could be greater than originally believed with up to 80% of the cases affected^5^. Although our study did not investigate the prevalence of *PTCH1* mutation, we found that the expression level of *PTCH1* in KCOT was decreased. The *PTCH1* gene functions as an important tumor suppressor^4^,^28^ which when suppressed, can lead to development of tumors. More importantly, the SHH pathway inhibitor Vismodegib has been used as an adjunctive therapy in patients with NBCCS to reduce tumor size and reduce the margins needed for surgical resection^29^. Currently, chemotherapeutic adjuncts are seldom used in sporadic cases; although they may be helpful in cases with decreased *PTCH1* expression in limiting tumor size, surgical margins and recurrence.

Although a large portion of the KCOT samples were FFPE which can result in RNA degradation^30^ and affect the performance of microarray analyses, previous studies supported the use of such samples^31^. Furthermore, NanoString analysis has been used for expression analysis in various sample types including fresh-frozen, FFPE and even whole cell lysates to produce excellent results^20^. Despite the issue of RNA degradation in FFPE samples, there was good correlation between the microarray and nanoString expression data for genes with the greatest fold changes. Another short-coming is the limited sample size of 20 tumors which is due to KCOT being a relatively rare tumor. However, it is comparable to other similar studies with sample sizes ranging from 10–12^12^,^13^. In addition, intra-tumor heterogeneity should be explored as the samples show inter-tumor heterogeneity^32^. Unfortunately, the cystic nature of KCOT means that epithelial cells were pooled from multiple slides and locations for each tumor to collect enough RNA for microarray analysis. The recent advent of single cell RNA-seq^33^ will allow future studies to conduct analysis at multiple locations of a single KCOT to examine possible differences in gene expression within each tumor in addition to between KCOT.

In conclusion, this study provides new insights into the transcriptome of KCOT using a method that provided purer sample populations of neoplastic epithelial cells. By comparing the KCOT transcriptome to the available “dentome”, we found familiar pathways that have been implicated in the formation of KCOT and other cancers. This is in addition to novel pathways that may serve as markers for diagnosis and prognosis. Possible targets for novel therapy were also identified in this study and should be investigated further to development precision medicine for the treatment of KCOT. Future studies should take into account the 2 distinct molecular subtypes when developing specific treatment modalities in order to maximize effectiveness.

## Additional Information

**How to cite this article**: Hu, S. *et al.* Transcriptome Variability in Keratocystic Odontogenic Tumor Suggests Distinct Molecular Subtypes. *Sci. Rep.* **6**, 24236; doi: 10.1038/srep24236 (2016).

## Supplementary Material

Supplementary Information

## Figures and Tables

**Table 1:** IPA canonical pathway analysis between the different KCOT molecular clusters and reference odontogenic tissue.

Canonical pathways differentially enriched (*p*<0.05) between KCOT “common tumor cluster” and normal cells				
Biological process	Ingenuity Canonical Pathways (z-score)			
Cell cycle regulation	Cell Cycle: G2/M DNA Damage Checkpoint Regulation	(−1.70)	Role of CHK Proteins in Cell Cycle Checkpoint Control	(−1.27)
	Aryl Hydrocarbon Receptor Signaling	(−1.63)	Antiproliferative Role of Somatostatin Receptor 2	(1.27)
	Role of BRCA1 in DNA Damage Response	(2.67)	Estrogen-mediated S-phase Entry	(1.13)
	Cyclins and Cell Cycle Regulation	(2.67)		
Inflammatory (Immune) response/cytokine signaling	Acute Phase Response Signaling	(2.20)	CXCR4 Signaling	(1.77)
	Extrinsic Prothrombin Activation Pathway	(1.13)	CD28 Signaling in T Helper Cells	(2.24)
	fMLP Signaling in Neutrophils	(2.29)	NRF2-mediated Oxidative Stress Response	(2.18)
Cellular growth, proliferation and apoptosis	Angiopoietin Signaling	(−1.39)	Oncostatin M Signaling	(2.33)
	Apoptosis Signaling	(1.09)	PEDF Signaling	(1.00)
	VEGF Signaling	(2.32)		
Cancer	Renal Cell Carcinoma Signaling	(1.73)	Pancreatic Adenocarcinoma Signaling	(1.61)
	Non-Small Cell Lung Cancer Signaling	(1.51)		
Tyrosine kinase signaling	Ephrin B Signaling	(−1.94)		
Others	Synaptic Long Term Depression	(1.10)		
**Canonical pathways differentially enriched (p<0.05) between KCOT “secretory ameloblast cluster” and normal cells**				
Cellular growth, proliferation and apoptosis	Paxillin Signaling	(−1.27)	Melanocyte Development and Pigmentation Signaling	(−1.00)
	Regulation of Cellular Mechanics by Calpain Protease	(−2.12)	PI3K/AKT Signaling	(1.67)
	Relaxin Signaling	(−1.00)	CREB Signaling in Neurons	(−1.27)
Inflammatory (Immune) response/cytokine signaling	IL-1 Signaling	(−1.41)	CXCR4 Signaling	(−1.16)
	fMLP Signaling in Neutrophils	(−1.00)	Macropinocytosis Signaling	(−1.00)
Cell cycle regulation	Antiproliferative Role of Somatostatin Receptor 2	(−1.34)	Integrin Signaling	(−1.21)
	Cell Cycle: G2/M DNA Damage Checkpoint Regulation	(1.00)		
G-protein signaling	Signaling by Rho Family GTPases	(−1.89)	Rac Signaling	(−1.90)
Tyrosine kinase signaling	Ephrin B Signaling	(−1.63)	Tec Kinase Signaling	(−1.90)
Cancer	Renal Cell Carcinoma Signaling	(−1.34)		
Others	GNRH Signaling	(−2.50)	Renin-Angiotensin Signaling	(−1.00)
	Cardiac Hypertrophy Signaling	(−1.21)	Agrin Interactions at Neuromuscular Junction	(−1.89)
**Canonical pathways differentially enriched (p<0.05) between KCOT “odontoblast cluster” and normal cells**				
Cellular growth, proliferation and apoptosis	IGF-1 Signaling	(1.51)	Oncostatin M Signaling	(1.34)
	HGF Signaling	(1.27)	Thrombopoietin Signaling	(1.63)
	NGF Signaling	(1.16)	FGF Signaling	(1.41)
	Melanocyte Development and Pigmentation Signaling	(2.53)	JAK/Stat Signaling	(1.89)
	AMPK Signaling	(1.94)	ErbB4 Signaling	(1.34)
	CREB Signaling in Neurons	(2.31)	PI3K/AKT Signaling	(1.00)
	Relaxin Signaling	(1.00)	EIF2 Signaling	(1.00)
	Regulation of eIF4 and p70S6K Signaling	(1.41)	Induction of Apoptosis by HIV1	(1.63)
Cell cycle regulation	Cell Cycle: G2/M DNA Damage Checkpoint Regulation	(−1.51)	Estrogen-mediated S-phase Entry	(1.34)
	Cyclins and Cell Cycle Regulation	(1.00)	Role of BRCA1 in DNA Damage Response	(2.24)
Cancer	Non-Small Cell Lung Cancer Signaling	(1.63)	Telomerase Signaling	(2.33)
	Glioma Signaling	(1.89)	Role of p14/p19ARF in Tumor Suppression	(−2.00)
Inflammatory (Immune) response/cytokine signaling	NRF2-mediated Oxidative Stress Response	(2.53)	IL-2 Signaling	(2.24)
	eNOS Signaling	(1.89)		
MAP kinase related	ERK/MAPK Signaling	(2.83)	UVB-Induced MAPK Signaling	(1.34)
	EGF Signaling	(2.83)		
Tyrosine kinase signaling	Neuregulin Signaling	(1.13)	Neurotrophin/TRK Signaling	(2.65)
G-protein signaling	Rac Signaling	(2.83)	PAK Signaling	(2.65)
Others	Renin-Angiotensin Signaling	(2.71)	Cardiac β-adrenergic Signaling	(1.67)
	GNRH Signaling	(2.50)	P2Y Purigenic Receptor Signaling Pathway	(1.90)
	Nitric Oxide Signaling in the Cardiovascular System	(2.12)	Prolactin Signaling	(1.13)

**Table 2:** Results of Gene set enrichment analysis (GSEA) conducted between KCOT and reference cells using “all curated gene sets v4.0”.

Gene sets enriched in KCOT “common tumor cluster” compared to normal cells				
Gene Set	Number of genes	Enrichment Score	Normalized enrichment score	Nominal p-value
SEKI_INFLAMMATORY_RESPONSE_LPS_UP	27	0.57	1.62	0.01
REACTOME_PLATELET_AGGREGATION_PLUG_FORMATION	10	0.66	1.66	0.01
JI_METASTASIS_REPRESSED_BY_STK11	10	0.70	1.42	0.02
HAHTOLA_SEZARY_SYNDROM_UP	38	0.51	1.39	0.02
SMID_BREAST_CANCER_RELAPSE_IN_BRAIN_UP	18	0.50	1.57	0.02
TONKS_TARGETS_OF_RUNX1_RUNX1T1_FUSION_GRANULOCYTE_UP	19	0.41	1.45	0.02
SMID_BREAST_CANCER_LUMINAL_A_DN	11	0.67	1.42	0.03
MOLENAAR_TARGETS_OF_CCND1_AND_CDK4_DN	34	0.68	1.40	0.03
BENPORATH_ES_CORE_NINE_CORRELATED	28	0.55	1.30	0.03
TAKEDA_TARGETS_OF_NUP98_HOXA9_FUSION_6HR_UP	32	0.42	1.39	0.04
ZHOU_TNF_SIGNALING_30MIN	11	0.57	1.46	0.04
SARTIPY_NORMAL_AT_INSULIN_RESISTANCE_UP	10	0.71	1.33	0.04
WAMUNYOKOLI_OVARIAN_CANCER_GRADES_1_2_UP	46	0.49	1.34	0.04
PID_AURORA_A_PATHWAY	13	0.64	1.55	0.05
FLOTHO_PEDIATRIC_ALL_THERAPY_RESPONSE_UP	10	−0.61	−1.56	0.03

**Table 3:** Results of IPA Upstream analysis indicating significantly (*p* < 0.05) differentially expressed molecules in KCOT clusters.

Differentially expressed molecules in KCOT “common tumor cluster”				
Molecule Type	Upstream Regulator (z-score)			
Transcription regulator	NUPR1	(−5.32)	NFE2L2	(2.59)
	MXI1	(−2.20)	FOXO1	(3.50)
	E2F6	(−2.65)	TP63	(2.61)
	KDM5B	(−4.57)	EZH2	(2.17)
	TP53	(−3.48)	MITF	(4.33)
	FOXL2	(2.65)	RUVBL1	(3.32)
	HIF1A	(2.93)	YAP1	(2.43)
	SP1	(2.17)	MYB	(2.59)
	JUN	(2.55)	RELA	(3.32)
	PPRC1	(3.06)	SMARCA4	(2.03)
	FOXM1	(3.33)		
Cytokine	IL4	(2.30)	IL1A	(3.83)
	CSF2	(3.86)	CCL5	(2.69)
	CD40LG	(2.02)	IL6	(3.81)
	IL17A	(2.24)	OSM	(2.28)
	TNF	(3.54)	IL1B	(3.32)
Kinase	STK17A	(−2.22)	EGFR	(3.10)
	PLK1	(−2.20)	MAP3K14	(2.43)
	TRIB3	(−2.65)	BRD4	(3.30)
	CDKN1A	(−3.28)	CCNK	(2.72)
Ligand-dependent nuclear receptor	PPARA	(2.20)	RARA	(3.45)
	PGR	(2.96)	ESR1	(3.71)
Enzyme	STUB1	(−2.63)	HRAS	(2.62)
	TRAF2	(2.97)	TERT	(2.07)
Complex	CD3	(−2.03)	NFkB (complex)	(3.34)
	IgG	(−2.23)	Cg	(4.54)
	I kappa b kinase	(−2.22)		
Group	STAT5a/b	(3.00)	ERK	(2.59)
	Jnk	(2.01)	Gm-csf	(2.16)
	E2f	(2.53)		
Growth factor	GDF2	(−2.00)	EGF	(2.81)
	FGF2	(2.20)		
Transporter	AZGP1	(−2.20)	SYVN1	(3.80)
Transmembrane receptor	TREM1	(3.72)	TNFRSF1A	(2.60)
Mature microrna	miR-34a-5p (and other miRNAs w/seed GGCAGUG)	(−2.46)	miR-122-5p (miRNAs w/seed GGAGUGU)	(−2.50)
Translation regulator	EIF4G1	(2.83)		
Peptidase	F7	(2.76)		
G-protein coupled receptor	HCAR2	(−2.12)		
Other	CBX7	(−2.24)	CD24	(4.00)
	PPP2R5C	(−2.33)	SELPLG	(2.31)
	UXT	(−2.88)	IGFBP2	(2.12)
	RABL6	(4.70)	RTKN	(2.00)
	HSPB2	(2.00)		
**Differentially expressed molecules in KCOT “secretory ameloblast cluster”**				
Complex	Cg	(2.41)		
Cytokine	CSF2	(2.36)		
**Differentially expressed molecules in KCOT “odontoblast cluster”**				
Transcription regulator	NUPR1	(−3.74)	TP53	(−3.06)
	SMARCE1	(−2.00)		
Kinase	CDKN1A	(−2.42)	MAPK9	(2.43)
