# Supplementary material for: Transcriptome Variability in Keratocystic Odontogenic Tumor Suggests Distinct Molecular Subtypes
Source: Sci Rep. 2016 Apr 12;6:24236. doi: 10.1038/srep24236 (PMC4828651; doi:10.1038/srep24236)
Supplement: Supplementary Information [file srep24236-s1.pdf]

Supplementary Information

Transcriptome Variability in Keratocystic Odontogenic Tumor Suggest Distinct Molecular Subtypes

Shijia Hu*[1,2], Kimon Divaris[1,3], Joel Parker[4], Ricardo Padilla[5], Valerie Murrah[5], John Timothy Wright[1]

[1]Pediatric Dentistry, University of North Carolina, Chapel Hill, NC, USA

[2]Faculty of Dentistry, National University of Singapore, Singapore

[3]Epidemiology, Gillings School of Global Public Health, University of North Carolina-Chapel Hill, Chapel Hill, NC, USA

[4]Cancer Genetics, University of North Carolina-Chapel Hill, Chapel Hill, NC, USA

[5]Diagnostic Sciences, School of Dentistry, University of North Carolina-Chapel Hill, Chapel Hill, NC, USA

Supplementary Fig. 1 – Scatterplot of Microarray differential expression versus NanoString differential expression. Microarray fold change is on the x-axis while NanoString fold change is on the y-axis.

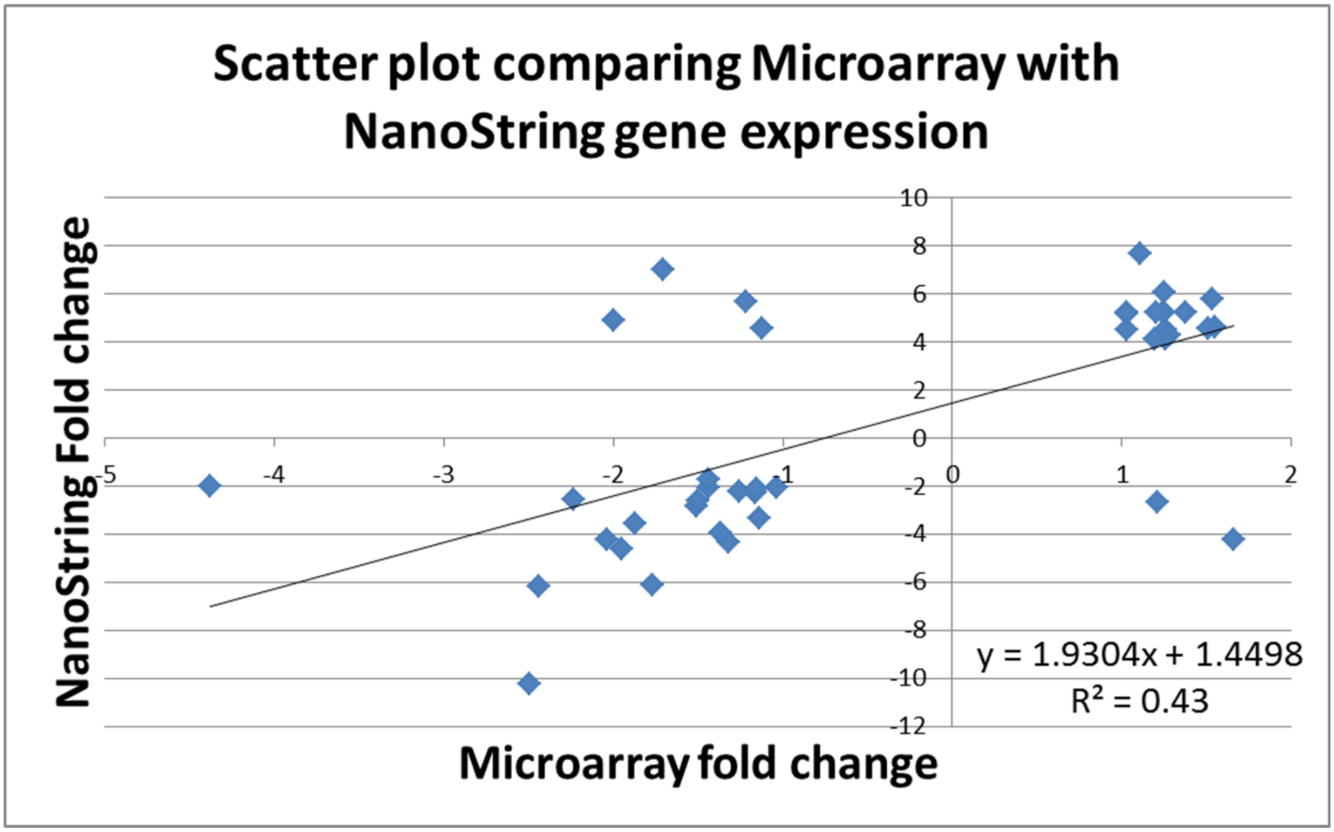

Supplementary Fig.2 – Micrograph of KCOT samples with the laser captured portions outlined in blue. A – Sample with absence of inflammation in the stroma. B – Sample with presence of inflammation in the stroma. The arrows show the presence of inflammatory cells.

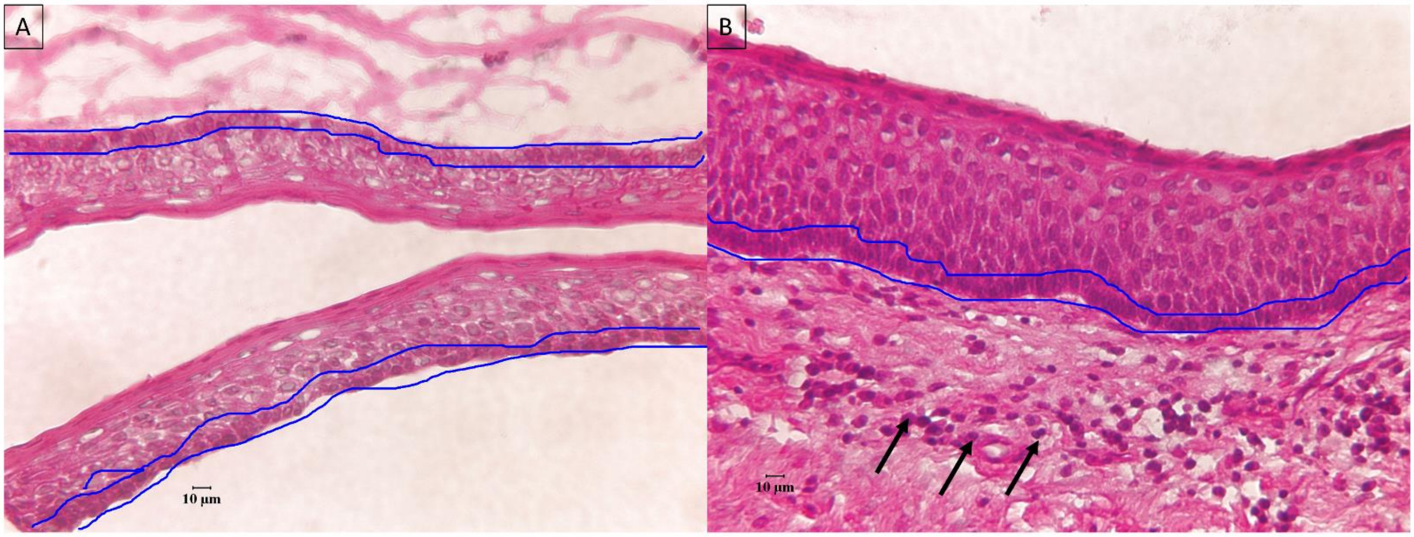

Supplementary Table 1: Nano string validation of microarray

Top 20 upregulated genes in KCOT compared to secretory ameloblast

Gene name	Nanostring fold change	Sam fold change
WT1	7.68	1.11
IL1B	7.01	-1.71
NTRK1	6.03	1.25
IL1A	5.80	1.54
CASP10	5.65	-1.22
AKT1	5.23	1.25
CYP1A1	5.23	1.37
FLT3	5.23	1.25
GATA1	5.23	1.03
TERT	5.23	1.21
CSF3	5.19	1.03
AREG	4.88	-2.00
TNF	4.59	1.55
PTGS2	4.55	-1.12
FGR	4.53	1.51
THPO	4.53	1.26
ATM	4.53	1.03
ABCB1	4.28	1.28
RET	4.14	1.26
EGF	4.10	1.19

Top 20 downregulated genes in KCOT compared to secretory ameloblast

Gene name	Nanostring fold change	sam fold change
SPP1	-10.26	-2.49
MMP2	-6.17	-2.44
PDGFRA	-6.12	-1.77
DLC1	-4.65	-1.95
FRZB	-4.33	-1.32
GAS1	-4.25	-2.04
IGFBP6	-4.25	1.66
CD34	-3.96	-1.37
ABL1	-3.57	-1.87
KDR	-3.37	-1.14
FGF2	-2.84	-1.51
LMO2	-2.70	1.21
ETS1	-2.60	-1.50
TEK	-2.58	-2.23
FYN	-2.29	-1.16
CCND2	-2.23	-1.26
GNAS	-2.13	-1.15
TGFBR3	-2.08	-1.03
FGFR1	-2.07	-1.44
CAV1	-2.01	-4.38
APC	-1.73	-1.44

Supplementary Table 2 - Genes in each cluster used for pathway analysis FDR < 1%

Data uploaded at http://genomewide.net/public/transcriptome/kcot/Supplemental_Table_2.xlsx
